# Gray Matter Changes in the Orbitofrontal-Paralimbic Cortex in Male Youths With Non-comorbid Conduct Disorder

**DOI:** 10.3389/fpsyg.2020.00843

**Published:** 2020-05-06

**Authors:** Yidian Gao, Yali Jiang, Qingsen Ming, Jibiao Zhang, Ren Ma, Qiong Wu, Daifeng Dong, Xiao Guo, Mingli Liu, Xiang Wang, Weijun Situ, Ruth Pauli, Shuqiao Yao

**Affiliations:** ^1^Medical Psychological Center of Second Xiangya Hospital, Central South University, Changsha, China; ^2^Medical Psychological Institute of Central South University, Changsha, China; ^3^China National Clinical Research Center on Mental Disorders (Xiangya), Changsha, China; ^4^Department of Psychiatry, The First Affiliated Hospital of Soochow University, Suzhou, China; ^5^Department of Radiology, Second Xiangya Hospital, Central South University, Changsha, China; ^6^Centre for Human Brain Health, School of Psychology, University of Birmingham, Birmingham, United Kingdom

**Keywords:** conduct disorder, comorbidities, gray matter volume, callous-unemotional traits, VBM

## Abstract

Conduct disorder is one of the most common developmental psychiatric disorders which is characterized by persistent aggressive and antisocial behaviors during childhood or adolescence. Previous neuroimaging studies have investigated the neural correlates underlying CD and demonstrated several constructive findings. However, Individuals with CD are at high risk for comorbidities, which might give rise to the inconsistencies of existed findings. It remains unclear which neuroanatomical abnormalities are specifically related to CD without comorbidities. Using structural magnetic resonance imaging (sMRI) data of 69 CD and 69 typically developing (TD) male youths (aged 14–17 years), the present study aims at investigating gray matter volume alterations of non-comorbid CD (i.e., not comorbid with attention deficit hyperactivity disorder, substance abuse disorder, anxiety or depression). We also examined how regional gray matter volumes were related to callous-unemotional (CU) traits and conduct problems in the CD group. The whole-brain analysis revealed decreased gray matter volumes in the right pre-postcentral cortex, supramarginal gyrus and right putamen in CD youths compared with TD youths. The region-of-interest analyses showed increased gray matter volumes in the superior temporal gyrus (STG) and right orbitofrontal cortex (OFC) in CD youths. Correlation analysis found that gray matter volume in the left amygdala was negatively correlated with CU traits in CD participants. These results demonstrated that gray matter volume in the orbitofrontal-paralimbic cortex, including OFC, STG and amygdala, might characterize the male youths with non-comorbid CD and might contribute to different severe forms and trajectories of CD.

## Introduction

Conduct disorder (CD) is one of the most common developmental psychiatric disorders (American Psychiatric Association [APA], 2013) which has serious negative influences on individuals, families and society (Moffitt et al., 1996). It is characterized by a repetitive and pervasive pattern of aggressive or antisocial behaviors during childhood and adolescence (American Psychiatric Association [APA], 2013). Children with CD are at increased risk for aggressive and antisocial behaviors that can potentially lead to serious offences, and mental and physical health problems in adulthood, making CD an important target of etiological research and prevention efforts (Moffitt et al., 2008; Dong et al., 2017; Fairchild et al., 2019).

Neural substrates abnormalities are important indicators of pathophysiological processes which can reflect disorder etiology (Stevens and Haney-Caron, 2012). In recent years, structural magnetic resonance imaging (sMRI) studies have identified several gray matter abnormalities in orbitofrontal, temporal, limbic and subcortical deficits in youths with CD (Fairchild et al., 2013; Zhang et al., 2014b; Jiang et al., 2015). Rogers and De Brito conducted a meta-analysis of sMRI studies on youths with conduct problems using voxel-based morphometry (VBM) methods (Rogers and De Brito, 2016). Based on 13 published VBM studies, they found decreased gray matter volumes in the left amygdala, right insula and left medial superior frontal gyrus and left fusiform gyrus (Rogers and De Brito, 2016). Another meta-analysis incorporating 12 sMRI and 17 fMRI studies of individuals with CD, with and without ADHD, reported reduced gray matter volumes in bilateral amygdala, bilateral insula, right striatum, left medial/superior frontal gyrus and left precuneus in individuals with CD (Noordermeer et al., 2016). Regarding structural deficits, prior work also reported some inconsistent findings. As an example, some studies provided evidence for higher gray matter concentration (Fairchild et al., 2013; Zhang et al., 2018) or no differences in the OFC (Sterzer et al., 2007; Bayard et al., 2018) whereas some studies reported reduced gray matter volumes in the OFC (Huebner et al., 2008; Fairchild et al., 2011; Stevens and Haney-Caron, 2012; Sebastian et al., 2016) when CD adolescents were compared with typically developing (TD) adolescents. Likewise, the amygdala, a key region for emotion processing (Blair, 2008), both positive (Stevens and Haney-Caron, 2012) and negative correlation (Sterzer et al., 2007) between CD symptoms and gray matter volumes in this region have been reported before.

Previous literature has indicated that these inconsistent findings might partially reflect the heterogeneity of the CD in the samples examined (Sterzer and Stadler, 2009). It has become increasingly clear that patients with CD are a highly heterogeneous population and may reflect distinct etiological pathways (Fanti, 2018). One of the main potential confounds of CD is comorbid psychopathology. Adolescents with CD are at high risk for multiple comorbidities, such as depression, anxiety disorder, substance abuse disorder (SUD) and attention-deficit/hyperactivity disorder (ADHD) (Nock et al., 2006; Connor et al., 2007). In this context, neuroimaging studies of CD are often confounded by comorbidities, especially CD with co-occurring ADHD (Beauchaine, 2012; Fanti, 2018). Although overlapping behaviorally, clinically and cognitively, CD youths with and without comorbidity may be affected by both similar and distinct underlying brain substrates (Olvera et al., 2014; Noordermeer et al., 2016). As suggested by Bayard et al. (2018), there are morphological characteristics that are specific to either ADHD symptoms or CD symptoms apart from the large overlap in structural relations (Bayard et al., 2018). Controlling for comorbidity as a potential confound can help clarify the etiology of CD (Fairchild et al., 2013). Given that, a few fMRI studies have investigated brain changes in youths with non-comorbid CD, demonstrating that the antisocial and aggressive behaviors exhibited by CD have consistently been associated with abnormalities in the ‘hot’ executive functions that involve incentives and motivation, and mediated by the orbitofrontal-paralimbic system, including orbitofrontal cortex (OFC), anterior cingulate cortex (ACC), superior temporal gyrus (STG) and underlying limbic cortex (Rubia et al., 2009a; Rubia et al., 2009b; Wu et al., 2017). However, most of the findings supporting ‘hot’ executive function hypothesis were reported by fMRI studies, meaning that far less is known about the neuroanatomical correlates of non-comorbid CD (Fahim et al., 2011; Rubia, 2011). To our knowledge, apart from one VBM study with mixed-gender samples (Stevens and Haney-Caron, 2012), no other sMRI study has directly examined brain structural abnormalities of non-comorbid CD boys. Given the lack of research on male CD youths, the primary aim of the study was to directly investigate the gray matter volume abnormalities in CD male youths without common comorbidities, such as ADHD, SUD, anxiety and depression. Another approach in the study of CD, particularly neuroimaging studies, has distinguished between CD children presenting with presence or absence of callous-unemotional (CU) traits (Frick and White, 2008). CU traits index low levels of empathy and guilt, and deficits in processing others’ fear and sadness (Frick and Viding, 2009; Blair, 2010). Previous studies have highlighted that high level of CU traits predict a more severe course and poorer health outcomes compared with non-CU CD groups (Viding et al., 2005; Frick and Viding, 2009). The influence of CU traits on neural substrates underlying CD has been observed by neuroimaging studies (De Brito et al., 2009; Jones et al., 2009; Passamonti et al., 2010; Fairchild et al., 2013; Cohn et al., 2016; Rogers and De Brito, 2016), although constructive findings have been reported, our knowledge about whether and how the CU traits are associated with brain structural changes in non-comorbid CD is still limited. Thus, the second aim of the present study was to investigate whether the variations of CU traits related to gray matter volume changes in CD male youths.

In this context, accounting for the heterogeneity of CD youths would help improve the reliability of CD-sMRI study findings. A better understanding of the neural correlates underlying non-comorbid CD may, in turn, help clarify the etiology of CD. Although several previous researchers have studied the heterogeneity in individuals with CD, with promising findings, the existing sMRI literature has some important limitations, including relatively small sample size and large age range. Thus, the current study used sMRI in conjunction with voxel-based morphometry (VBM) to examine gray matter abnormalities in non-comorbid, non-medicated CD boys, in a relatively large sample size (69 CD and 69 TD boys) and within a small age range (14–17 years old). In line with previous studies, we expected to find gray matter volume alterations in several regions in the CD group, especially the regions involved in the ‘hot’ executive functions, such as OFC, STG and limbic brain regions (Rubia, 2011; Rogers and De Brito, 2016). We also hypothesized that gray matter volumes in anterior insula and amygdala would be negatively correlated with the CU traits (Blair, 2007; Cohn et al., 2016). The gray matter volumes in the putamen and orbital-frontal cortex (OFC) would be positively correlated with CU traits (Fairchild et al., 2013; Rogers and De Brito, 2016).

## Materials and Methods

### Participants

Given that CD rates are greater in males and to remove gender as a confounding variable (Giedd et al., 1997; Fairchild et al., 2013; Zhang et al., 2014a; Cao et al., 2018), this study enrolled only boys. A total of seventy male adolescents with non-comorbid CD (aged 14–17 years old) were recruited from outpatient clinics affiliated with the Second Xiangya Hospital of Central South University in Changsha, Hunan, China. A TD group of seventy-three age-, gender-, and IQ-matched volunteers was recruited from regular secondary schools. All participants and their parents were made aware of the purpose of the study and gave written informed consent for participation.

The inclusion period for CD and TD subjects was between November 2011 to August 2018 (see Supplementary Material for the recruitment flow-charts). The seventy boys who met criteria for the non-comorbid CD were selected from a total sample of one hundred and eighty CD adolescents. Compared to included CD participants, excluded CD participants also meet ADHD diagnoses. All participants underwent an independent structured clinical interview by two well-trained psychiatrists. Diagnoses of CD were made based on the widely used Structured Clinical Interview for the DSM-IV-TR Axis I Disorders-Patient Edition (SCID-I/P) (First et al., 1997; First et al., 2005), which has been shown to be valid and reliable in China (Phillips and Liu, 2011; Tiansheng Guo et al., 2014). During the diagnostic interview, all participants were assessed for CD using the SCID-I/P user guide. Psychiatrists rated each CD symptom item as (0) absent, (1) subclinical or (2) clinically present. The assessment of age-of-onset of CD symptom and medication status was also included in the interview. Age-of-onset was defined as the first time when CD symptom or functional impairment was present, as reported by participants or their caregivers (American Psychiatric Association [APA], 2013). Diagnostic interviews with participants and caregivers were carried out separately. The psychiatrists made the final decision whether the information obtained from participants and caregivers was inconsistent. A further interview was conducted to get more information when there were any inconsistencies between the information provided by the participant and the caregiver. All patients recruited met the DSM-IV-TR criteria for CD. None had a history of psychotropic medication treatment before or during participation in the study (treatment-naïve). The exclusion of ADHD followed the same procedures with diagnoses of CD based on the interviews using SCID-I/P.

For TD group recruitment, male students who matched the CD subjects’ ages were recruited through flyers in the local middle schools. Volunteers were interviewed by the same psychiatrists and subjected to the SCID-I/P. Information provided by TD subjects was verified by their parents on an as-needed basis. None of the TD participants met the criteria for CD. Moreover, the exclusion criteria for both groups were assessed in diagnostic interviews. The exclusion criteria included: history of ADHD, or any other behavioral disorder; history of any psychiatric or emotional disorder; the presence of any pervasive developmental or chronic neurological disorder (e.g., autism), Tourette’s syndrome, post-traumatic stress disorder, obsessive-compulsive disorder; persistent headaches, head trauma; alcohol or SUD in the past year; contraindications to MRI; or a full-scale IQ < 80, as estimated by the Wechsler Intelligence Scale for Children-Chinese revision (C-WISC) examinations (Gong and Cai, 1993).

### Measures

The Chinese version of the Center for Epidemiologic Studies Depression Scale (CES-D) (Radloff, 1991) and the Multidimensional Anxiety Scale for Children (MASC) (March et al., 1997; Yao et al., 2007) were used to rate depression and anxiety severity, respectively. The Strengths and Difficulties Questionnaire (SDQ) (Goodman, 2001) was used to measure the conduct problem and ADHD symptom (i.e., hyperactivity and inattention), which exhibited high levels of reliability and validity in Chinese adolescents (Yao et al., 2009). The CU subscale of the Antisocial Process Screening Device (APSD) was used to assess CU traits and other associated psychopathic traits (Vitacco et al., 2003). The self-report scores on the APSD showed moderate stability across 1–2 years and significant correlations with measures of antisocial behavior both concurrently and predictively (Munoz and Frick, 2007). All of the subjects were right-handed according to the Edinburgh Handedness Inventory (Oldfield, 1971).

### Image Acquisition

Images were acquired by a Philips Achieva 3-T scanner (Amsterdam, Netherlands) with a standard head coil at the Medical Imaging Department in Second Xiangya Hospital of Central South University (Changsha, China). MRI data were acquired with *T*_1_-weighted three-dimensional magnetization-prepared rapid gradient-echo sequences with following acquisition parameters: voxel size = 1 × 1 × 1 mm^3^, repetition time = 8.5 ms, echo time = 3.743 ms, flip angle = 8°, acquisition matrix = 256 × 256 pixels, field of view = 256 × 256, number of slices = 180, slice thickness = 1 mm.

### Image Processing

#### Quality Control

All images were visually inspected before processing for scanner artifacts and gross neuroanatomical abnormalities, such as tumors or cysts. After image pre-processing, the Computational Anatomy Toolbox (CAT12, http://www.neuro.uni-jena.de/cat/) provided ratings of image data quality, which were used to identify problems with individual images. These ratings assess basic image properties, noise and geometric distortions (e.g., due to motion) and combine them into a weighted image quality rating (IQR). All of the scans rated B and above in IQR, representing good image quality. Finally, an automated quality check using covariance analysis on the sample homogeneity of segmented gray matter images was performed in CAT12 toolbox (Gaser and Dahnke, 2016). Five participants (four TD and one CD) were excluded due to motion artifacts, leaving sixty-nine participants for each group in the final sample.

#### Pre-processing

Images were cropped and reoriented following the anterior-posterior commissure line with MRIcro^1^. The resulting whole-brain images were processed with SPM12^2^ in Matlab 2017a (Math-works, Natick, MA, United States); the anterior commissure was set manually in each image as the origin of the spatial coordinates. The Template-O-Matic toolbox was used to create a standardized *a priori* tissue probability map (TPM) based on the age of the 138 participants (Wilke et al., 2008). The high-resolution T1-weighted scans were segmented with reference to the TPMs into gray matter and white matter images using the CAT12 by means of voxel-based morphometry (VBM) (Gaser and Dahnke, 2016). Diffeomorphic Anatomical Registration Through Exponentiated Lie algebra (DARTEL) toolbox was used to import the segmented gray and white matter images into a native space, create a template from the merged images of the 138 subjects, and to warp, modulate, normalize, and smooth the individual results using an 8-mm full-width at half-maximum (FWHM) isotropic Gaussian kernel (Ashburner, 2007). The template was normalized to MNI and registered to MNI (ICBM) space. The total intracranial volume (TIV) for each participant was calculated and used as a covariate for further statistical analyses.

### Statistical Analyses

Following preprocessing, MRI statistical analyses were performed in SPM12 using a general linear model to permit quantification of group effects, after controlling for potentially confounding covariates: TIV, age and IQ. Two approaches were applied for group comparison. First, for whole-brain analysis, a significance threshold at *P* < 0.05 was used with voxel-level familywise error (FWE) rate correction for multiple comparisons to protect against type I errors (Worsley et al., 1996; Friston, 1997). For the region of interest (ROI) analyses, results were reported at *P* < 0.05, small volumes correction (SVC) (Worsley et al., 1996; Friston, 1997). Prior ROIs, including the bilateral anterior insula, amygdala, superior temporal gyrus (STG) and orbitofrontal cortex (OFC) and were defined as ROIs using Automatic Anatomical Labeling (AAL) (Tzourio-Mazoyer et al., 2002) ROIs from the Wake Forest University (WFU) Pickatlas (Maldjian et al., 2003) given their critical roles in the pathophysiologic mechanism of CD (Rubia, 2011; Fairchild et al., 2013; Noordermeer et al., 2016; Rogers and De Brito, 2016).

Besides, we conducted exploratory whole-brain regression analysis between gray matter volume and CU traits in CD group only (controlled for TIV, age, IQ, conduct problem scores and age-of-onset). Regression analysis was also used to investigate the relationship between conduct problem scores (controlled for TIV, age, IQ, CU traits and age-of-onset) with gray matter volumes in CD group. Results were thresholded at *P* < 0.05, voxel-level FWE correction. Where significant results were found, contrast estimates from the peak voxel were extracted to assess the direction of effects in SPSS 19.0 and plot the results.

## Results

### Demographic and Clinical Variables

The demographic and clinical characteristics of the subjects are summarized in Table 1. No significant differences were detected in age, IQ, depression/anxiety severity, SDQ emotion problem or hyperactivity/inattention subscale. Relative to TD youths, CD youths reported higher levels of self-reported conduct problem and CU traits, and endorsed lower levels of peer problem and prosocial behavior (Table 1).

**TABLE 1:** Demographic and clinical characteristics of study participants.

	Group		Analysis	
		
Variables	Typically Developing (mean ± SD)	Conduct Disorder (mean ± SD)	*t*	*P* value
Age	15.40.64	15.10.96	1.76	0.08
IQ	95.6113.26	92.8612.65	1.25	0.21
CES-D scores	15.678.87	13.519.67	1.37	0.17
MASC scores	37.8114.87	38.4618.46	–0.23	0.82
**SDQ scores**				
*emotion symptom*	2.862.09	3.452.58	–1.49	0.14
*conduct problem*	3.161.91	4.142.05	–2.90	**0.004**
*hyperactivity/inattention*	4.201.57	4.622.16	–1.31	0.19
*peer problem*	4.031.73	3.351.88	2.22	**0.028**
*prosocial behavior*	7.301.74	6.002.18	3.89	**<0.001**
**APSD scores**				
*CU traits*	2.601.63	3.832.72	–3.21	**0.002**
*total scores*	10.453.58	14.345.07	–5.09	**<0.001**
TIV (cm^3^)	1596.15105.22	1597.71114.87	–0.08	0.93

*SD = Standard Deviation; IQ = Intelligence Quotient; CES-D = Chinese Version of the Center for Epidemiologic Studies Depression Scale; MASC = Multidimensional Anxiety Scale for Children; SDQ = The Strengths and Difficulties Questionnaire; APSD = Antisocial Process Screening Device; CU = Callous-unemotional Traits; TIV = Total Intracranial Volume.*

### Neuroimaging

#### Gray Matter Volume Alterations Between Groups

**FIGURE 1:**
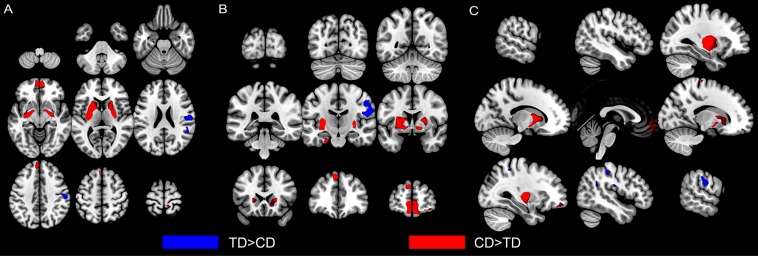
Altered gray matter volumes in male youths with non-comorbid conduct disorder (CD) compared with typically developing (TD) male youths. Group comparison of GMVs revealed that CD youths showed significant decreased gray matter volumes (in blue) in the right postcentral gyrus (extending to supramarginal gyrus) and superior temporal gyrus than TD youths. CD youths also showed increased gray matter volumes (in red) in left putamen and left orbitofrontal cortex. Regional gray matter differences were shown from axial **(A)**, coronal **(B)**, and sagittal **(C)** view. The image is thresholded at *P* < 0.001, uncorrected for display purposes. CD = Conduct Disorder; TD = Typically Developing.

There were no significant group differences in mean estimated TIV (Table 1). The whole-brain analysis revealed significantly reduced gray matter volume of the right pre-postcentral gyrus, extending to right supramarginal gyrus in the CD group, compared to those in the TD group (Table 2, Figure 1). Meanwhile, the CD group showed increased gray matter volume of the left putamen in comparison with the TD group. ROI analysis revealed decreased right STG volume (peak voxel = [51, −39, 23], *k* = 37, *P* = 0.038, *Z* = 3.56, SVC) and increased left OFC volume (peak voxel = [−2, 54, −20], *k* = 8, *P* = 0.027, *Z* = 3.40, SVC) in the CD group when compared with TD group. We did not find any other significant gray matter volume differences between groups elsewhere in the brain (additional data are given in the Supplementary Material).

**TABLE 2:** Whole-brain analysis results showing significant differences between typically developing youths and conduct disorder youths.

		MNI Coordinates					
		
Contrast	Hemisphere	x	y	z	SDM *z* value	*P*^a^	No. of Voxels
***CD* < *TD***							
Pre-postcentral gyrus, extending to supramarginal gyrus	Right	53	−14	24	4.59	**0.029**	815
		59	−21	35	3.70		
		56	−20	45	3.51		
***CD* > *TD***							
Putamen	Left	−29	−15	5	4.82	**0.011**	63

*MNI = Montreal Neurological Institute; SDM = Signed Differential Mapping; CD = Conduct Disorder; TD = Typically Developing; OFC = Orbitofrontal Cortex. ^*a*^Voxel-probability threshold: *P* < 0.05 (whole brain, voxel-level family wise error correction).*

#### Correlations Between Gray Matter Volumes With CU Traits in CD Participants

**FIGURE 2:**
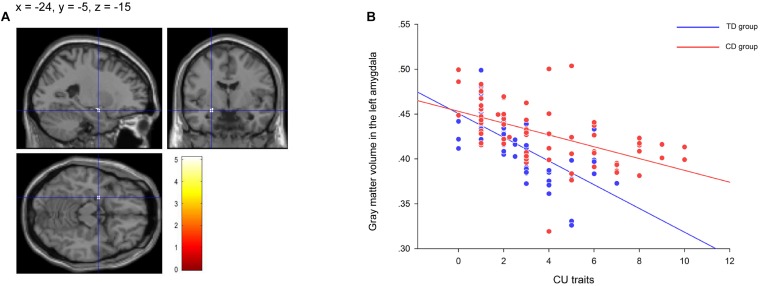
The significant correlation between gray matter volume in the left amygdala and callous-unemotional (CU) traits in the conduct disorder (CD) group and the typically developing (TD) group. The negative correlation between CU traits and gray matter volumes in the left amygdala (−24, −5, −15) **(A)** showing in the residual plot **(B)** within the CD participants and within the TD participants after controlling for total intracranial volume, age, IQ, conduct problem scores and age-of-onset. The color bar represents *t* statistics. The image is thresholded at *P* < 0.05, whole-brain voxel-level family wise error correction. CD = Conduct Disorder; TD = Typically Developing.

Gray matter volumes in the left amygdala (peak voxel = [–24, –5, –15], *k* = 30, *Z* = 4.66, *P*_*FWE*_ = 0.027) was negatively correlated with CU traits in CD participants (Figure 2A). The correlation was significant when controlling for conduct problem scores in SDQ (*r* = −0.662, *P* < 0.001; Figure 2B), suggesting that the left amygdala volume was more strongly related to variations of CU traits instead of CD symptoms. Further analysis revealed that the gray matter volume in the left amygdala was also negatively correlated with CU traits in TD participants (*r* = –0.543, *P* < 0.001; Figure 2B). No positive correlation was detected in the brain.

#### Correlations Between Gray Matter Volumes With Conduct Problems in CD Participants

The whole-brain analysis did not detect any brain regions that showed a correlation with conduct problem scores for SDQ in the CD group (*P*_*FWE*_ > 0.05). However, a positive correlation between gray matter volume in right OFC (peak voxel = [12, 51, –21], *k* = 93, *Z* = 3.59) and conduct problem scores was observed at a more liberal significance threshold (*P*_*uncorrected*_ < 0.001). No other correlation was detected in the brain.

## Discussion

The present study was the largest single study to compare gray matter volume in male youths with non-comorbid CD and matched TD male youths. In line with our first hypothesis, altered gray matter volume detected in CD participants were mostly located within brain regions involved in the ‘hot’ executive functions (Rubia, 2011). The whole-brain analysis revealed reduced gray matter volume of the right pre-postcentral gyrus (extending to right supramarginal gyrus) and increased gray matter volume of the left putamen in the CD group when compared with the TD group. Meanwhile, ROI analysis revealed reduced right STG volume and increased left OFC volume in the CD group when compared with the TD group. Supporting our second hypothesis, we also observed a negative correlation between CU traits and gray matter volumes in the left amygdala, which is crucial for empathy and emotion processing (Hillis, 2018; Marsh, 2018).

Previous studies have found that emotion regulation and motivation recruited lateral orbitofrontal, ventromedial frontal, superior temporal, and limbic brain regions (Davidson et al., 2000; Blair, 2008; Rubia, 2011). Our finding of altered gray matter volumes in temporal (i.e., STG) and frontostriatal (i.e., OFC and putamen) cortex matched the cortical topography of the ‘hot’ execution functioning theory, which provided evidence that deficits of the orbitofrontal-temporal structures may be specifically associated with non-comorbid CD (Rubia, 2011). Gray matter volume and cortical thickness reductions in the STG have been reported in previous studies on CD adolescents (Hyatt et al., 2012; Jiang et al., 2015; Rogers and De Brito, 2016) and criminal psychopaths (Müller et al., 2008). The STG has been implicated in many aspects of affective behavior and social abilities, including empathy (Leslie et al., 2004; Hooker et al., 2010), affective processing (Kiehl, 2006) and moral reasoning (Raine and Yang, 2006). According to an fMRI study, the STG also played an important role in inferring and predicting the mental and emotional states of others (Sebastian et al., 2012a; Sebastian et al., 2012b; Xiong et al., 2019). Given that STG plays a critical role in empathy and has been found structurally changed in CD youths (Hyatt et al., 2012; Jiang et al., 2015), the consonance between fMRI, sMRI studies and our findings highlight that deficits in the STG may contribute to the neurocognitive abnormalities exhibited by CD adolescents.

The OFC, which is included in the ‘hot’ orbitofrontal-paralimbic system (Rubia, 2011), has been demonstrated to play a critical role in reward and punishment processing (O’doherty, 2004), recognition of angry expressions (Blair and Cipolotti, 2000) and reactive aggression (Blair, 2012). Along with ACC, OFC mediates top-down affect regulation via its interconnection with underlying limbic areas (Davidson et al., 2000; Rubia, 2011). In addition, several human lesion studies and fMRI work in healthy participants have identified that the OFC, together with other regions, is one of the core regions for human moral cognition (Murray et al., 2007). Lesions in OFC may lead to disadvantageous consequences such as violations of social norms (Séguin, 2004). Altered gray matter volume in the OFC has been consistently observed by previous VBM studies of CD youths (De Brito et al., 2009; Fairchild et al., 2013; Sebastian et al., 2016). However, the results related to the OFC volume alterations remain inconsistent. As prior studies reported, gray matter concentration was increased in the medial OFC in boys with CU and conduct problems (De Brito et al., 2009; Fairchild et al., 2013). Some studies reported reduced OFC volume in CD youths, but most of the clusters reported were located in the lateral part of OFC (Huebner et al., 2008; Rogers and De Brito, 2016; Sebastian et al., 2016). It should be noted that mounting findings have emphasized that the OFC is a functionally heterogeneous region, suggesting that the lateral or ventromedial area of OFC may play different roles in stimulus-based learning and decision making (McClure et al., 2004; McClure et al., 2007; Mar et al., 2011; Izquierdo, 2017). Several studies suggested that the lateral OFC is involved in the evaluation of options (Walton et al., 2010; Rudebeck and Murray, 2011), whereas the medial OFC is more important for comparing and selecting options (Rudebeck and Murray, 2011; Hunt et al., 2012). Our finding suggested that the structural alterations in OFC region exhibited by CD adolescents could be heterogeneous, and the increased ventromedial OFC volume may underlie deficits in reward processing and decision-making of CD adolescents without ADHD (Matthys et al., 2013).

Apart from the OFC, we also detected increased gray matter volume in the putamen, a region associated with inhibitory control and reinforcement learning, which refer to “cool” and “hot” executive functions respectively (O’doherty et al., 2004; Di Martino et al., 2008). The finding was consistent with previous sMRI studies that reported increased putamen volume in antisocial individuals (Barkataki et al., 2006; Schiffer et al., 2011) and positive correlations between putamen volume and CU traits in different populations (Glenn et al., 2010; Fairchild et al., 2011; Rogers and De Brito, 2016). These findings suggest that increased volumes in the putamen are related to the antisocial features as well as CU traits. However, inconsistent findings were also reported (Fairchild et al., 2011; Aoki et al., 2013; Fairchild et al., 2013). As demonstrated in Fairchild *et al.* (2013), the reduced striatal volume effect was non-significant when factoring out ADHD symptoms or excluding participants with comorbid ADHD. This result is consistent with studies showing that ADHD is associated with reduced volume in the putamen (Ellison-Wright et al., 2008; van Wingen et al., 2013). Taken together, the inconsistent findings found in the putamen may be contributed by the ADHD comorbidity. The observed gray matter abnormalities in the frontostriatal cortex might account for the impairments in regulating social behavior and processing socio-emotional stimuli exhibited by CD youths.

Interestingly, the whole-brain analysis also detected decreased gray matter volume in the right pre-postcentral gyrus (extending to supramarginal gyrus) in CD participants. Although we did not predict this finding, the result is consistent with previous studies on adolescents with conduct problems (Dalwani et al., 2015; Rogers and De Brito, 2016) and male CD adolescents (Smaragdi et al., 2017) reporting gray matter volume reductions in same regions. Both of the postcentral gyrus and the supramarginal gyrus are important parts in the somatosensory association cortex, which is traditionally thought to be involved in spatial localization (Ungerleider, 1982) and the control of action (Goodale and Milner, 1992). However, recent neuroimaging studies observed that the postcentral gyrus, supramarginal gyrus and the STG have also been involved in the mirror neuron system (MNS), offering a potential neural mechanism for automatically imitating other people’s actions and understanding their motor plan (Molenberghs et al., 2009; Rizzolatti and Sinigaglia, 2010). Substantial evidence suggested that the MNS might be an important neurobiological contributor to emotional contagion as well as empathic processing (Schulte-Rüther et al., 2007; Van Overwalle and Baetens, 2009), since the MNS allows us to resonate with the emotional state of others with a simulation mechanism (Krautheim et al., 2019; Rymarczyk et al., 2019). Given the important role in empathy, the significant gray matter volume reductions in the right postcentral gyrus, the supramarginal gyrus as well as the STG might provide preliminary evidence for structural deficits in MNS underlying abnormal empathic processing exhibited by CD adolescents.

The present study observed robust negative associations between CU traits and gray matter volumes in the left amygdala in CD participants, which is consistent with previous fMRI studies of CD demonstrating a negative correlation between CU traits and amygdala activity (Jones et al., 2009; Viding et al., 2012). CU traits are linked to shallow affect, lack of empathy and other affective deficiencies in youth (Frick and White, 2008). Adolescents with elevated CU traits seem to exhibit deficits in experiencing empathic arousal to fear and distress in others and abnormalities in responses to cues of danger and punishment (Frick and White, 2008). Notably, the left amygdala which showed a negative correlation with CU traits has been found to play a critical role in empathy and emotion processing (Blair, 2008; Fan et al., 2011). Therefore, the impaired capacity of empathy of youths who exhibit higher CU traits may be related to gray matter volumes in the left amygdala.

## Limitations

We noted several limitations of the present study. Firstly, the age-of-onset data were based on retrospective reports, which might be associated with recall bias. To minimize the effect of recall bias, we collected data from both participants and their caregivers. The psychiatrist would ask for more details and make the final judgment if the information provided was inconsistent. Secondly, the clinical characteristics of the subjects were based on self-perspective data. For CD participants with tendencies to manipulate, deceive or minimize faults, the validity of self-report assessment may be compromised (Kelley et al., 2016). Inconsistent with previous findings, the study observed a lower level of peer problems exhibited by CD participants, which might due to highly positively biased answers given by CD participants. Thus, replication is needed to provide further support for the present study with multiple sources of information (i.e., parent-report and teacher-report assessment). Finally, the present study was cross-sectional, so our findings cannot be used to make definite causal claims of a cause-and-effect relationship between antisocial behaviors and CD-associated brain structural abnormalities. The present findings can be extended by future studies examining similar issues in participants from birth cohorts or using longitudinal designs.

## Conclusion

To our knowledge, this is the largest single VBM study to investigate the gray matter volume alterations of CD boys with consideration of CU traits and age-of-onset. In conclusion, the present study replicated the gray matter volume aberrations in supramarginal gyrus, STG and OFC and extended these findings to male youths with non-comorbid CD. The results further suggested critical roles for the orbitofrontal-paralimbic cortex, including OFC, STG and amygdala in affecting empathy and moral judgment in ways that contribute to observable psychopathological symptoms of CD.

## Data Availability Statement

The datasets generated for this study are available on request to the corresponding author.

## Ethics Statement

The studies involving human participants were reviewed and approved by the Ethics Committee of the Second Xiangya Hospital of Central South University (No. CSMC-2009S167). Written informed consent to participate in this study was provided by the participants’ legal guardian/next of kin.

## Author Contributions

SY and XW: study concept and design. WS and SY: study supervision. YG, YJ, QM, JZ, DD, QW, and XG: acquisition, analysis, or interpretation of the data. YG: drafting of the manuscript. SY and WS: administrative, technical, or material support. All authors: critical revision of the manuscript for important intellectual content.

## Conflict of Interest

The authors declare that the research was conducted in the absence of any commercial or financial relationships that could be construed as a potential conflict of interest.

## Footnotes

1 http://www.cabiatl.com/mricro/mricro/mricro.html

2 http://www.fil.ion.ucl.ac.uk/spm
