# Supplementary material for: Gray Matter Changes in the Orbitofrontal-Paralimbic Cortex in Male Youths With Non-comorbid Conduct Disorder
Source: Front Psychol. 2020 May 6;11:843. doi: 10.3389/fpsyg.2020.00843 (PMC7218112; doi:10.3389/fpsyg.2020.00843)
Supplement: Supplementary file 1 [file Data_Sheet_1.doc]

# Gray matter changes in the orbitofrontal-paralimbic cortex in male youths with non-comorbid conduct disorder

Yidian Gao^1,2,3^, Yali Jiang^1,2,3^, Qingsen Ming^4^, Jibiao Zhang^1,2,3^, Ren Ma^1,2,3^, Qiong Wu^1,2,3^, Daifeng Dong^1,2,3^, Xiao Guo^1,2,3^, Mingli Liu^1,2,3^, Xiang Wang^1,2,3^, Weijun Situ^5^, Ruth Pauli^6^, Shuqiao Yao^1,2,3*^

^1^ Medical Psychological Center of Second Xiangya Hospital, Central South University, Changsha, Hunan, China

^2^ Medical Psychological Institute of Central South University, Changsha, Hunan, China

^3^ China National Clinical Research Center for Mental Disorders (Xiangya), Changsha, Hunan, China

^4^ Department of Psychiatry, the First Affiliated Hospital of Soochow University, Suzhou, Jiangsu, China

^5^ Department of Radiology, Second Xiangya Hospital, Central South University, Changsha, Hunan, China

^6^ Centre for Human Brain Health, School of Psychology, University of Birmingham, Birmingham, UK

*** Correspondence:**

Professor. Shuqiao Yao

[shuqiaoyao@csu.edu.cn](mailto:shuqiaoyao@csu.edu.cn)

**Supplementary materials**

Table S1. Brain regions showing differences between typically developing group and conduct disorder group survived at whole-brain *P* < .001, uncorrected.

| Contrast | Hemisphere | MNI Coordinates | | | SDM  *z* value | *P*^a^ | No. of  Voxels |
| --- | --- | --- | --- | --- | --- | --- | --- |
|  |  | x | y | z |  |  |  |
| ***CD < TD*** |  |  |  |  |  |  |  |
| Pre-postcentral gyrus, ext. supramarginal gyrus | Right | 53 | -14 | 24 | 4.59 | < .001 | 815 |
|  |  | 59 | -21 | 35 | 3.70 |  |  |
|  |  | 56 | -20 | 45 | 3.51 |  |  |
| STG | Right | 51 | -42 | 26 | 3.82 | < .001 | 157 |
| Inferior parietal gyrus | Right | 30 | -42 | 53 | 3.50 | < .001 | 8 |
| Postcentral gyrus | Right | 45 | -29 | 48 | 3.31 | < .001 | 96 |
| ***CD > TD*** |  |  |  |  |  |  |  |
| Putamen | Left | -29 | -15 | 5 | 4.82 | < .001 | 1557 |
|  |  | -18 | -6 | -8 | 4.21 |  |  |
|  |  | -15 | 5 | 6 | 3.59 |  |  |
| OFC, ext. vmPFC | Left | -5 | 62 | -20 | 4.27 | < .001 | 909 |
|  |  | -2 | 63 | -5 | 4.02 |  |  |
| Putamen | Right | 29 | -12 | 3 | 4.20 | < .001 | 627 |
|  |  | 11 | 0 | -6 | 3.89 |  |  |
| dmPFC | Left | -5 | 59 | 32 | 4.14 | < .001 | 111 |
| Uncus | Left | -18 | -17 | -33 | 3.91 | < .001 | 204 |

*Note: MNI = Montreal Neurological Institute; SDM = Signed Differential Mapping; TD= Typically Developing; CD = Conduct Disorder; STG = Superior Temporal Gyrus; OFC =* *Orbitofrontal Cortex; vmPFC = Ventromedial Prefrontal Cortex ; dmPFC = Dorsomedial Prefrontal Cortex; ext. = extending to.*

^a^ Voxel-probability threshold: *P* < .001, whole brain uncorrected.

Table S2. Regional gray matter volumes showing significant correlation with callous-unemotional traits in conduct disorder participants survived at whole-brain *P* < .001, uncorrected

|  | Hemisphere | MNI Coordinates | | | SDM  *z* value | *P*^a^ | No. of  Voxels |
| --- | --- | --- | --- | --- | --- | --- | --- |
|  |  | x | y | z |  |  |  |
| Amygdala, ext. hippocampus and putamen | Left | -24 | -5 | -15 | 4.66 | <.001 | 1519 |
|  |  | -27 | -20 | -3 | 3.70 |  |  |
|  |  | -26 | -9 | 5 | 3.21 |  |  |
| Postcentral gyrus, ext. superior temporal gyrus | Left | -53 | -11 | 15 | 4.44 | <.001 | 708 |
|  |  | -66 | -14 | 18 | 3.65 |  |  |
| Lingual gyrus, ext. cerebellum | Left | -3 | -65 | -5 | 4.43 | <.001 | 465 |
|  |  | -9 | -72 | -5 | 4.28 |  |  |
| Cerebellum | Right | 50 | -60 | -26 | 4.20 | <.001 | 396 |
| Inferior frontal gyrus | Right | 54 | 29 | 3 | 3.82 | <.001 | 158 |

*Note: MNI = Montreal Neurological Institute; SDM = Signed Differential Mapping; ext. = extending to.*

^a^ Voxel-probability threshold: *P* < .001, whole brain uncorrected.

**Recruitment flow-chart for the CD group**

Total screening for CD group

(N = 510)

Participants met criterion for CD by SCID-I/P

(N = 180)

**Excluded due to**

1. History of ADHD, or any other behavioral disorder ( N = 81);

2. Hstory of any psychiatric or emotional disorder, the presence of any pervasive developmental or chronic neurological disorder (e.g., autism), Tourette's syndrome, post-traumatic stress disorder, obsessive-compulsive disorder ( N = 9);

3. Contradictions for MRI ( N = 2);

5. Alcohol or substance use in the past year ( N = 3);

6. History of psychotropic medication treatment ( N = 3);

8. Left-handedness (N = 6);

9. IQ < 80 (N = 6);

**Excluded due to**

excessive head motion (N = 1)

CD participants included for analysis

(N = 69)

CD participants included for scanning

(N = 70)

**Recruitment flow-chart for the TD group**

Total screening for TD group

(N = 87)

Participants did not met criterion for CD by SCID-I/P

(N = 82)

**Excluded due to**

1. History of ADHD, or any other behavioral disorder ( N = 4);

2. Hstory of any psychiatric or emotional disorder, the presence of any pervasive developmental or chronic neurological disorder (e.g., autism), Tourette's syndrome, post-traumatic stress disorder, obsessive-compulsive disorder ( N = 0);

3. Contradictions for MRI ( N = 1);

5. Alcohol or substance use in the past year ( N = 0);

6. History of psychotropic medication treatment ( N = 0);

8. Left-handedness (N = 3);

9. IQ < 80 (N = 1);

10. Age, IQ not matched with CD participants ( N = 2 )

**Excluded due to**

excessive head motion (N = 4)

TD participants included for analysis (N = 69)

TD participants included for scanning

(N = 73)
